# Dynamic regulation of anthocyanin biosynthesis at different light intensities by the BT2-TCP46-MYB1 module in apple

**DOI:** 10.1093/jxb/eraa056

**Published:** 2020-01-30

**Authors:** Jian-Ping An, Ya-Jing Liu, Xiao-Wei Zhang, Si-Qi Bi, Xiao-Fei Wang, Chun-Xiang You, Yu-Jin Hao

**Affiliations:** 1 State Key Laboratory of Crop Biology, Shandong Collaborative Innovation Center for Fruit and Vegetable Production with High Quality and Efficiency, College of Horticulture Science and Engineering, Shandong Agricultural University, Tai-An, Shandong, China; 2 CONICET- National University of La Plata, Argentina

**Keywords:** Anthocyanin accumulation, apple, high-light intensity, *Malus domestica*, post-transcriptional regulation, TCP transcription factor

## Abstract

Teosinte branched1/cycloidea/proliferating (TCP) transcription factors play a broad role in plant growth and development, but their involvement in the regulation of anthocyanin biosynthesis is currently unclear. In this study, anthocyanin biosynthesis induced by different light intensities in apple (*Malus domestica*) was found to be largely dependent on the functions of the MdMYB1 and MdTCP46 transcription factors. The expression of MdTCP46 was responsive to high light intensity, and under these conditions it promoted anthocyanin biosynthesis by direct interactions with MdMYB1 that enhanced the binding of the latter to its target genes. MdTCP46 also interacted with a bric-a-brac/tramtrack/broad (BTB) protein, MdBT2, that is responsive to high light intensity, which ubiquitinated MdTCP46 and mediated its degradation via the 26S proteasome pathway. Our results demonstrate that the dynamic regulatory module MdBT2-MdTCP46-MdMYB1 plays a key role in modulating anthocyanin biosynthesis at different light intensities in apple, and provides new insights into the post-transcriptional regulation of TCP proteins.

## Teaser

An apple TCP transcription factor, MdTCP46, is induced by high light intensity and interacts with MdMYB1 to promote expression of anthocyanin biosynthesis genes.

## Introduction

Anthocyanins are a type of water-soluble pigment that are found in plant flowers, fruits, stems, leaves, and seeds. In recent decades, anthocyanin biosynthesis has been studied extensively due to the important antioxidant properties of these compounds and their effects on the appearance of commercial plant produce. Anthocyanins are synthesized through the phenylpropanoid pathway, in which a series of enzymes play key roles, including dihydroflavonol 4-reductase (DFR), UDP flavonoid glucosyl transferase (UF3GT), chalcone isomerase (CHI), and chalcone synthase (CHS) (Winkel-Shirley, 1999; Hichri *et al.*, 2011). It is well established that anthocyanin biosynthesis is regulated by the WD40-bHLH-MYB complex at the transcription level through the direct mediation of the expression of its biosynthetic genes (Carbone *et al.*, 2009; Hichri *et al.*, 2011). External stimuli, such as temperature, light, water, nutrients, and exogenous hormones, also play key roles in the regulation of anthocyanin biosynthesis (Winkel-Shirley, 2001, 2002; Carbone *et al.*, 2009; Jaakola, 2013; Honda and Moriya, 2018).

Light is one of the most important environmental factors regulating anthocyanin biosynthesis (Takos *et al.*, 2006; Jaakola, 2013). Numerous studies have demonstrated that light stimulates the accumulation of anthocyanin and that its biosynthesis does not occur in the dark (Meng *et al.*, 2004; Azuma *et al.*, 2012; Zoratti *et al.*, 2014; Guan *et al.*, 2016; Jiang *et al.*, 2016). The light quality also affects biosynthesis, especially ultraviolet and blue light (Ordidge *et al.*, 2012; Henry-Kirk *et al.*, 2018; Tao *et al.*, 2018; Zhang *et al.*, 2018*a*). Studies on apple, strawberry, pear, and peach have shown that ultraviolet and blue light treatments promote the expression of transcription factors (TFs) such as MYB, BBX, ERF, and HY5, which in turn activate the expression of anthocyanin biosynthetic genes and ultimately lead to increased anthocyanin levels (Ubi *et al.*, 2006; Gong *et al.*, 2015; Henry-Kirk *et al.*, 2018; Zhang *et al.*, 2018*a*; An *et al.*, 2019*a*; Fang *et al.*, 2019; Ni *et al.*, 2019). Light intensity also affects anthocyanin biosynthesis (Jaakola, 2013; Trojak and Skowron, 2017; Zhang *et al.*, 2018*b*). Previous studies have suggested that TCP and MYB TFs may play roles in anthocyanin accumulation and may be mediated by conditions of different light intensity (Takos *et al.*, 2006; Viola *et al.*, 2016).

In many species, MYB1 and its orthologs (MYB10 and MYBA) play key roles in the regulation of anthocyanin biosynthesis, and they are known to be positive regulators of accumulation that is mediated by various environmental and hormone signaling pathways (Takos *et al.*, 2006; Ban *et al.*, 2007; Espley *et al.*, 2007; Lin-Wang *et al.*, 2010; Niu *et al.*, 2010; Telias *et al.*, 2011; Schwinn *et al.*, 2016; Yi *et al.*, 2018). In apple (*Malus domestica*), overexpression of *MdMYB1* promotes accumulation by activating the transcripts of anthocyanin biosynthetic genes such as *MdDFR* and *MdUF3GT* (Takos *et al.*, 2006; Ban *et al.*, 2007; Espley *et al.*, 2007). Previous studies have demonstrated that MdMYB1 can coordinate with MdbHLH3, MdERF3, MdbZIP44, MdWRKY40, and MdERF38 to regulate the biosynthesis of anthocyanin that is promoted by low temperature, ethylene, abscisic acid (ABA), wounding, and drought stress, respectively (Xie *et al.*, 2017; An *et al.*, 2018*c*, 2018*d*, 2019*c*, 2020). In addition, MdMYB1 undergoes ubiquitination and degradation, that is mediated by MdCOP1 and MdBT2 in response to multiple hormonal and environmental signals (Li *et al.*, 2012; Wang *et al.*, 2018).

The teosinte branched1/cycloidea/proliferating (TCP) family encodes specific TFs that feature a TCP domain with a bHLH motif (Cubas *et al.*, 1999; Manassero *et al.*, 2013; Nicolas and Cubas, 2016). In Arabidopsis, 24 TCP proteins have been identified and categorized into two classes based on the presence of the TCP motif (Cubas *et al.*, 1999; Martín-Trillo and Cubas, 2010; Manassero *et al.*, 2013). TCP family proteins have also been discovered in other plant genomes, including rice and apple (Yao *et al.*, 2007; Xu *et al.*, 2014). In apple, they are organized into three classes based on differences in their sequences (Xu *et al.*, 2014). As TFs, TCP proteins regulate the expression of target genes by binding to specific promoter sequences [e.g. GGNCCCAC, GGNCC, GCCCR, or G(T/C)GGNCCC; Aggarwal *et al.*, 2010). TCP proteins have also been documented to function by directly interacting with a variety of proteins, including other TCPs (Danisman *et al.*, 2012), MYB (Li and Zachgo, 2013), NAC (Weir *et al.*, 2004), and bZIP proteins (Mukhopadhyay and Tyagi, 2015).

There is evidence that TCP proteins play broad roles in plant growth and development, including cell proliferation, gametophyte development, seed germination, leaf and flower development, branching, regulation of the circadian clock, and multiple hormone signals (Corley *et al.*, 2005; Tatematsu *et al.*, 2008; Guo *et al.*, 2010; Koyama *et al.*, 2010; Martín-Trillo and Cubas, 2010; Pruneda-Paz and Kay, 2010; Kieffer *et al.*, 2011; Sarvepalli and Nath, 2011; Braun *et al.*, 2012; Min *et al.*, 2015; Busch *et al.*, 2019). Although the functions and mechanisms of TCP proteins have been studied extensively, there has been limited examination of their roles in the integration of plant development and environmental signals. Recent studies have shown that TCP15 responds to conditions of high light intensity in Arabidopsis (Viola *et al.*, 2016). In rice, OsTCP19 has been found to positively regulate responses to salt and water-deficit stresses synergistically with OsABI4 (Mukhopadhyay and Tyagi, 2015).

Several TCP proteins have been found to regulate the biosynthesis of secondary metabolites. In Arabidopsis, TCP3 promotes ﬂavonoid accumulation by interacting with the MYB proteins that regulate anthocyanin biosynthesis (Li and Zachgo, 2013). TCP15 acts as a negative regulator in biosynthesis of anthocyanins that is modulated by high light (Viola *et al.*, 2016). In *Artemisia annua*, AaTCP14 activates the expression of *DBR2* and *ALDH1*, two key genes of artemisinin biosynthesis (Ma *et al.*, 2018). In *Lycium ruthenicum*, LrTCP4 has been found to play a key role in the synthesis of kukoamine A (Chahel *et al.*, 2019). Although the regulatory mechanisms of TCP proteins that are involved in plant growth and development through the direct transcriptional regulation of target genes or interaction with other proteins have been widely studied, this is not the case for their transcriptional and post-transcriptional regulation mechanisms. There are just a few studies that have shown that TCP proteins may be regulated by miR319 (Schommer *et al.*, 2014; Bresso *et al.*, 2018; Palatnik and Weigel, 2019, Preprint).

In the present study, we found that MdTCP46 acted as a positive regulator of anthocyanin biosynthesis induced by high light intensity in apple. *MdTCP46* expression was induced and degradation of the protein was delayed after treatment with high light intensity. MdTCP46 was also found to interact with MdMYB1, and this was essential for anthocyanin biosynthesis induced at high light intensity. MdTCP46 enhanced the binding activity of MdMYB1 to its target gene promoters. In addition, MdTCP46 directly interacted with MdBT2, which is a negative modulator of anthocyanin biosynthesis. The expression of MdBT2 was suppressed by high light intensity at both the transcriptional and post-translational levels. MdBT2 suppressed the role of MdTCP46 by inducing its ubiquitination, resulting in subsequent degradation via the 26S proteasome pathway. Our findings uncover a potential regulatory mechanism of anthocyanin biosynthesis in apple based on the functioning of the BT2-TCP46-MYB1 protein module under different light intensities.

## Materials and methods

### Plant material and treatments

Uncolored fruit of apple (*Malus domestica*) ‘Red Delicious’ at 120 d after full bloom were collected from mature trees in an orchard located in Tai’an (Shandong, China). To examine the effects of different light intensities on anthocyanin accumulation in the peel, the fruit were divided into four groups and stored in incubators at 22 °C under different light conditions. The first group was stored with no light. The other groups were stored with 16/8 h photoperiods at either low light (~80 μmol m^–2^ s^–1^), moderate light (~150 μmol m^–2^ s^–1^), or high light (~300 μmol m^–2^ s^–1^). The fruit were treated for 3–6 d and images were taken. Fruit treated for 1 d were used for gene expression analysis. The peel was detached by hand using a peeler.

Calli of the variety ‘Orin’ and detached leaves of tissue-cultured seedlings of the variety ‘GL-3’ were used for genetic transformation and for determination of the effects of different light intensities on anthocyanin accumulation. The growing conditions of the calli and apple seedlings were as previously described (An *et al.*, 2018*b*, 2019*b*).

### Plasmid construction

The ORFs of *MdTCP3*, *MdTCP12*, *MdTCP21*, and *MdTCP46* were fused to pGAD424 to generate *MdTCP3*-pGAD, *MdTCP12*-pGAD, *MdTCP21*-pGAD, and *MdTCP46*-pGAD. *MdMYB1* with the autonomously activated domain deleted was fused to pGBD to generate *MdMYB1*^*△*^-pGBD. *MdBT2* and its N- and C-terminal fragments were fused to pGBT9 to generate *MdBT2*-pGBD, *MdBT2*-N-pGBD, and *MdBT2*-C-pGBD. The ORFs of *MdTCP46*, *MdMYB1*, and *MdBT2* were fused to pET32a or pGEX 4T-1 to generate *MdTCP46*-pGEX4T-1, *MdTCP46*-pET32a, *MdMYB1*-pET32a, and *MdBT2*-pGEX4T-1. The ORFs of *MdTCP46*, *MdMYB1*, and *MdBT2* were fused to YFP^N^ or YFP^C^ to generate *MdTCP46*-YFP^N^, *MdTCP46*-YFP^C^, *MdMYB1*-YFP^C^, and *MdBT2*-YFP^N^.

To generate constructs for the dual luciferase assays, the ORFs of *MdTCP46* and *MdMYB1* were inserted into pGreenII62-SK. The promoter sequences of *MdDFR* and *MdUF3GT* were cloned into pGreenII0800-LUC.

The ORF of *MdTCP46* was inserted into IL60-2 and TRV2 to generate MdTCP46-IL60-2 and MdTCP46-TRV2, respectively. IL60-1 and TRV1 acted as auxiliary plasmids. The MdTCP46 transient overexpression construct (MdTCP46-pIR) consisted of IL60-1 and MdTCP46-IL60-2. And the MdTCP46 transient antisense construct (MdTCP46-TRV) consisted of TRV1 and MdTCP46-TRV2.

To generate MdTCP46-overexpression (-OX) lines, the ORFs of *MdTCP46* were fused to pCXSN-GFP. To generate *MdBT2*-OX, the ORFs of *MdBT2* were fused to pRI101. To generate *MdTCP46*-antisense (-Anti), *MdMYB1*-Anti, and *MdBT2*-Anti, sequences of *MdTCP46*, *MdMYB1*, and *MdBT2* were inserted into the pCXSN vector. All primers are listed in Supplementary Table S3 at *JXB* online.

### Genetic transformation

Transgenic apple calli, leaves, and fruit, and Arabidopsis were obtained as previously described (An *et al.*, 2018*b*, 2019*c*). In brief, wild-type (WT) apple calli were incubated with *Agrobacterium* carrying recombinant plasmids and genetically transformed calli were screened on selective media (An *et al.*, 2018*b*, 2019*c*). Transgenic Arabidopsis seedlings were obtained by the floral dip method (Clough and Bent, 1998). Transient transgenic apple leaves were generated by vacuum infiltration (An *et al.*, 2018*b*, 2019*c*).

### Apple injection assays

The overexpression viral vector *MdTCP46*-IL60-2 was generated by inserting the CDS of *MdTCP46* into the IL60-2 vector. The IL60-1 vector was used as an auxiliary plasmid. The antisense viral vector *MdTCP46*-TRV2 was obtained by cloning the CDS of *MdTCP46* into the TRV2 vector. The TRV1 vector was used as an auxiliary plasmid. The TRV vectors were introduced into *Agrobacterium tumefaciens* LBA4404. The mixed vectors and the *A. tumefaciens* solutions were injected into the fruit peels.

### Measurement of anthocyanin contents

Anthocyanin was extracted using an extraction buffer containing anhydrous ethanol and hydrochloric acid as previously described (An *et al.*, 2018*c*, 2019*c*). In brief, samples were placed in the extraction buffer for 5 h. After centrifugation, the supernatant was collected, and its absorption values at 530, 620, and 650 nm were determined using a spectrophotometer (Soptop, Shanghai, China) (An *et al.*, 2018*c*, 2019*c*).

### Quantitative real-time PCR

RNA extraction was performed using a RNAplant plus Reagent (Tiangen) as previously described (An *et al.*, 2018*a*, 2020). Quantitative real-time PCR (qRT-PCR) was conducted to identify transgenic material and to detect *MdTCP46* and *MdBT2* transcripts. To determine the light responses of *MdTCP46* and *MdBT2*, fruit of ‘Red Delicious’ were sampled at 120 d after full bloom and kept in bags in the dark before being placed in the different light-intensity conditions. Three biological replicates and three technical replicates were used in all qRT-PCR reactions. All primers are listed in Supplementary Table S3.

### Yeast two-hybrid assays

The pGAD424 and pGBT9 vectors (Clontech) were used to perform yeast two-hybrid (Y2H) assays. The pGAD424 vector contained a GAL4 activation domain, and the pGBT9 vector contained a GAL4 binding domain. The ORFs of the *MdTCP46* and *MdMYB1* sequences lacking the autonomously activated fragments were fused to the pGAD424 and pGBT9 vectors, respectively. *MdTCP3*-pGAD, *MdTCP12*-pGAD, *MdTCP21*-pGAD, *MdTCP46*-pGAD, *MdMYB1*^*△*^-pGBD, *MdBT2*-pGBD, *MdBT2*-N-pGBD, and *MdBT2*-C-pGBD were constructed as described above. The genetic transformation of Y2H Gold yeast cells (Clontech) was accomplished by PEG induction. Transformed yeast cells were cultured on a selective medium for 3 d, and Y2H assays were performed as previously described (An *et al.*, 2019*b*, 2020). The pGAD424 and pGBT9 empty vectors were used as negative controls.

### Pull-down assays

The pET32a and pGEX4T-1 vectors (Novagen) were used to perform pull-down assays. The pET32a vector contained a HIS tag, and pGEX4T-1 contained a GST tag. *MdTCP46*-pGEX4T-1, *MdTCP46*-pET32a, *MdMYB1*-pET32a, and *MdBT2*-pET32a were constructed as described above. The fusion proteins of MdTCP46-GST, MdTCP46-HIS, MdMYB1-HIS, and MdBT2-HIS were obtained by isopropyl β-D-1-thiogalactopyranoside (IPTG) induction. A polyhistidine binding resin pull-down kit (Pierce™ His Protein Interaction Pull-Down Kit, ThermoFisher Scientific) was used in this study. GST proteins were used as negative controls. The eluted solution was treated with HIS and GST antibodies (Abmart, Shanghai, China) and pull-down assays were performed as previously described (An et al., 2019*b*, 2020).

### Bimolecular fluorescence complementation assays

The pSPYNE-35S/pUC-SPYNE and pSPYCE-35S/pUC-SPYCE vectors (Clontech) were used to perform bimolecular fluorescence complementation (BiFC) assays. The pSPYNE-35S/pUC-SPYNE vector contained a YFP^N^ domain, and the pSPYCE-35S/pUC-SPYCE vector contained a YFP^C^ domain. *MdTCP46*-YFP^N^, *MdTCP46*-YFP^C^, *MdMYB1*-YFP^C^, and *MdBT2*- YFP^N^ were constructed as described above. Onion epidermis was incubated with an *Agrobacterium* solution carrying the relevant plasmid and the cells were observed 3 d later using a FV3000 fluorescence microscope (Olympus). BiFC assays were performed as previously described (An *et al.*, 2019*b*, 2020).

### Screening of proteins interacting with MdMYB1

An apple gene library and the *MdMYB1*^*△*^-pGBD plasmid were prepared to screen MdMYB1-interacting proteins using a Y2H system, as previously described (An *et al.*, 2018*d*). Four potential interacting proteins were obtained, namely MdTCP3 (accession no. MDP0000243495), MdTCP12 (MDP0000173048), MdTCP21 (MDP0000851695), and MdTCP46 (MDP0000319941). Given the false-positive phenomenon of Y2H screening, the interactions were further confirmed through one-to-one Y2H assays. Only MdTCP46 was found to be a direct interacting protein with MdMYB1.

### Electromobility shift assays

Electromobility shift assays (EMSAs) were conducted to determine the effects of MdTCP46 on the binding activity of MdMYB1 to the promoters of *MdDFR* and *MdUF3GT*. MdTCP46-HIS and MdMYB1-HIS fusion proteins were obtained by IPTG-mediated induction. The probe was labeled with biotin by the Sangon Biotech Co. (Shanghai, China). Designated proteins and probes were mixed in the binding buffer for 25 min. The binding and free probes were separated by acrylamide gel, and EMSAs were performed as previously described (An *et al.*, 2018*d*)

### Dual-luciferase assays

The pGreenII62-SK and pGreenII0800-LUC vectors were used to perform dual-luciferase assays. The pGreenII62-SK vector contained a 35S promoter, and the pGreenII0800-LUC vector contained a LUC tag. *MdTCP46*-pGreenII62-SK, *MdMYB1*-pGreenII62-SK, *proMdDFR*-pGreenII0800-LUC, and *proMdUF3GT*-pGreenII0800-LUC were constructed as described above. The plasmid combinations were injected into the back of a leaf of *Nicotiana benthamiana* by *Agrobacterium*-mediated transformation. The pGreenII62-SK and pGreenII0800-LUC empty vectors were used as negative controls. Fluorescence activity was detected using a fluorescence detection kit (Promega) (An *et al.*, 2018*d*).

### Protein degradation assays *in vitro*

To determine the effects of different light intensities on the stability of the MdTCP46 and MdBT2 proteins, fruit of ‘Red Delicious’ (120 d after full bloom) were subjected to the low- and high-light treatments for 3 d. The peels were collected, and total proteins were extracted and incubated with the MdTCP46-GST and MdBT2-HIS fusion proteins. Samples were taken at 0–6 h of incubation and protein residues were determined using HIS and GST antibodies (Abmart).

To determine the effects of MdBT2 on the stability of the MdTCP46 protein, WT and transgenic apple calli extracts were incubated with the MdTCP46-GST protein. Samples were taken at 0–6 h of incubation and protein residues were determined using a GST antibody (Abmart). Protein degradation assays *in vitro* were performed as previously described (An *et al.*, 2019*b*, 2020). Briefly, calli were ground in liquid nitrogen, soaked with protein extraction liquid, and the supernatant was obtained following centrifugation.

To examine the effects of the MG132 proteasome inhibitor, total proteins from calli were pre-treated with 100 µM MG132 dissolved in DMSO for 0.5 h. DMSO alone was used as the blank control.

### Detection of protein ubiquitination *in vivo*

Detection of protein ubiquitination *in vivo* was performed using a Pierce™ Co-Immunoprecipitation Kit (ThermoFisher Scientific). In brief, the MdTCP46-GFP protein was extracted from apple calli using a GFP antibody (Abmart) and immunoprecipitated proteins were examined using ubiquitin and GFP antibodies (Abmart). Protein ubiquitination detection *in vivo* was performed as previously described (An *et al.*, 2019*c*).

### Statistical analyses

All experiments were conducted three times with consistent results, and data are presented for one representative experiment. Experimental results were analysed using the DPS v7.05 software by one-way ANOVA and LSD *post hoc* tests.

### Accession numbers

Sequence data in this study can be obtained from the apple gene function and gene family (https://gfdb.sdau.edu.cn; https://www.rosaceae.org/node/1), TAIR (https://www.arabidopsis.org/), and NCBI (https://www.ncbi.nlm.nih.gov/) databases under the following accession numbers: *MdTCP3* (MDP0000243495), *MdTCP12* (MDP0000173048), *MdTCP46* (MDP0000319941), *MdTCP21* (MDP0000851695), *MdBT2* (MDP0000151000), *MdMYB1* (MDP0000259614), *MdDFR* (MDP0000494976), and *MdUF3GT* (MDP0000405936).

## Results

### MdMYB1 acts as a positive regulator of anthocyanin accumulation under high light intensity

It has been demonstrated that light promotes anthocyanin accumulation in apple, strawberry, pear, and peach (Ubi *et al.*, 2006; Gong *et al.*, 2015; Henry-Kirk *et al.*, 2018; Zhang *et al.*, 2018*b*; An *et al.*, 2019*a*; Fang *et al.*, 2019; Ni *et al.*, 2019). In this study, uncolored ‘Red delicious’ apple fruit were stored in incubators under different conditions of light intensity. Compared to storage under constant dark conditions, fruit consistently turned red after treatment with light, and higher light intensities clearly up-regulated the expression of genes related to anthocyanin biosynthesis, resulting in higher contents (Supplementary Fig. S1A–C).

MdMYB1 is known to be involved in anthocyanin biosynthesis, and overexpression of *MdMYB1* contributes to its accumulation in apple (Takos *et al.*, 2006; Ban *et al.*, 2007; Espley *et al.*, 2007). We constructed a *MdMYB1*-antisense suppression plasmid and transformed it into apple calli (Supplementary Fig. S2A). In the wild-type (WT) plants, moderate and high light intensities progressively and significantly increased anthocyanin accumulation compared to low-light conditions (Supplementary Fig. S1D, E). In contrast, *MdMYB1*-Anti plants showed no difference between low and moderate light, and only a relatively small increase in accumulation under high-light conditions. These results indicated that MdMYB1 was essential for the induction of anthocyanin biosynthesis under high light intensity.

### MdTCP46 interacts with MdMYB1

We conducted a search for proteins that interact with MdMYB1 in a yeast screening library. The MdMYB1 protein with the autonomously activated domains removed was fused to pGBT9 to generate MdMYB1^△^-pGBD. An apple TCP protein was obtained (An *et al.*, 2018*b*) and a NCBI BLAST search showed it to be a TCP15-like protein (Supplementary Fig. S3). We named it as MdTCP46, according the systematic classification of the apple TCP family (Xu *et al.*, 2014).

**Fig. 1.**
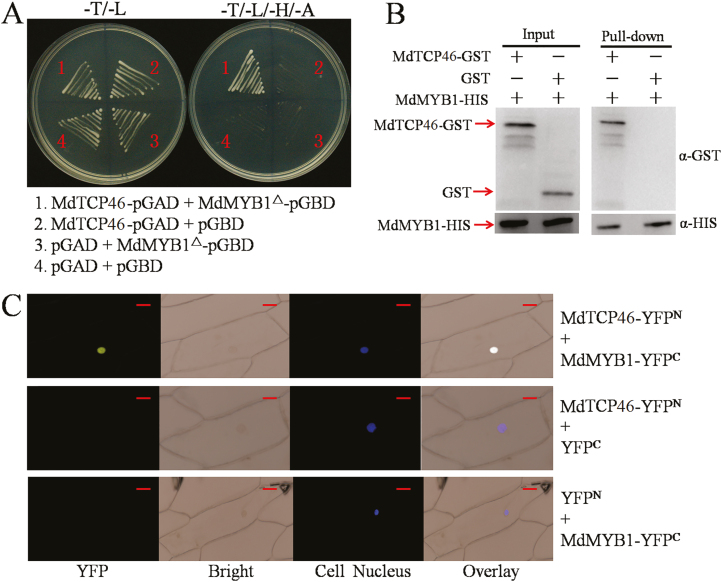
Apple MdTCP46 interacts with MdMYB1. (A) Yeast two-hybrid assays. The ORFs of the *MdTCP46* and *MdMYB1* sequences lacking the autonomously activated fragments were fused to the pGAD424 and pGBT9 vectors, respectively. The pGAD424 and pGBT9 empty vectors were used as negative controls. –T/–L, SD medium lacking Trp and Leu; –T/–L/–H/–A, SD medium lacking Trp, Leu, His, and Ade. (B) Pull-down assays. The ORFs of *MdMYB1* and *MdTCP46* were fused to the pET32a and pGEX4T-1 vectors, respectively. The MdMYB1-HIS and MdTCP46-GST fusion proteins were obtained from *E. coli* expression. GST proteins were used as negative controls. The eluted solution was probed using HIS and GST antibodies. (C) Bimolecular fluorescence complementation assays. The ORFs of *MdTCP46* and *MdMYB1* were fused to the YFP^N^ and YFP^C^ vectors, respectively. Fluorescence was examined using a confocal laser-scanning microscope. Scale bars are 10 μm.

The physical interaction between MdTCP46 and MdMYB1 was confirmed by three different assays (Fig. 1). First, *MdTCP46*-pGAD and *MdMYB1*^*△*^-pGBD were constructed and transformed into Gold yeast cells for Y2H assays. Only yeast cells with both MdTCP46 and MdMYB1 grew normally on selective medium (–T/–L/–H/–A) (Fig. 1A), suggesting a direct interaction. Second, fusion proteins were prepared for pull-down assays and the results indicated that MdTCP46-GST was pulled-down by MdMYB1-HIS (Fig. 1B). Third, BiFC assays were developed that linked *MdTCP46* with the N-terminus of YFP (*MdTCP46*-YFP^N^) and *MdMYB1* with the C-terminus of YFP (*MdMYB1*-YFP^C^). The resulting fluorescence signals showed that MdTCP46 interacted with MdMYB1 in the nucleus (Fig. 1C). Collectively, these data indicated that MdTCP46 physically interacts with MdMYB1.

### MdTCP46 promotes anthocyanin biosynthesis

**Fig. 2.**
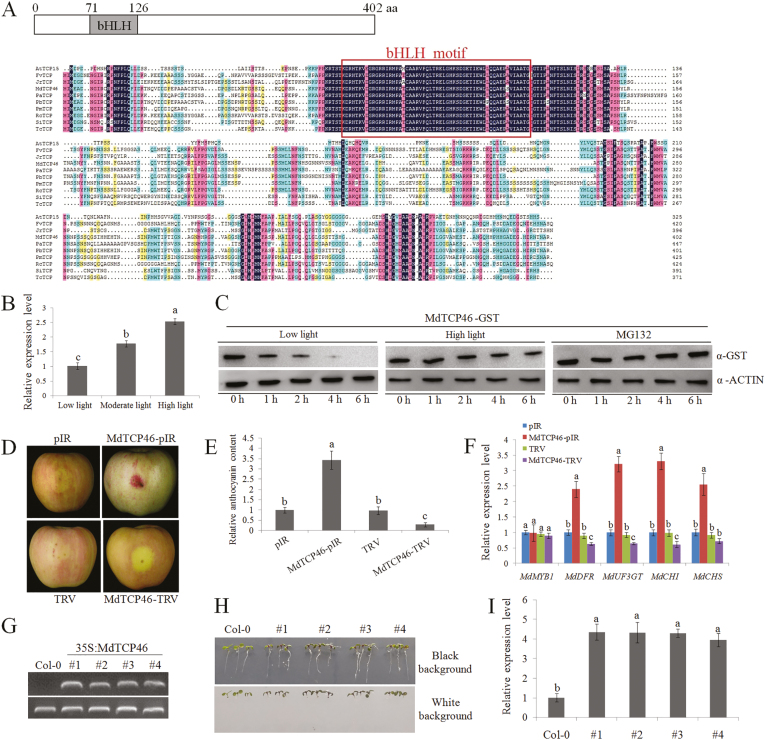
Overexpression of *MdTCP46* promotes anthocyanin biosynthesis in apple. (A) Multiple alignment of the bHLH domain in 10 TCP proteins obtained from the NCBI database. PbTCP, *Pyrus* × *bretschneideri* (XP_009360352.1); PmTCP, *Prunus mume* (XP_008221641.1); RcTCP, *Rosa chinensis* (XP_024194958.1); FvTCP, *Fragaria vesca* (XP_004297311.1); PaTCP, *Parasponia andersonii* (PON70380.1); JrTCP, *Juglans regia* (XP_018817718.1); SiTCP, *Sesamum indicum* (XP_011071008.1); TcTCP, *Theobroma cacao* (XP_017969954.1); MdTCP46, *Malus* × *domestica* (MDP0000319941); AtTCP15, *Arabidopsis thaliana* (AT1G69690.1). The bHLH motif is indicated. (B) Transcription of *MdTCP46* under different light intensities using qRT-PCR. Plants were treated for 3 d and expression was normalized to the *actin* gene. Values are relative to the low-light treatment, which was set as 1. (C) Detection of the MdTCP46-GST fusion protein after dark and moderate light treatments. ‘Red Delicious’ apple fruit were treated with low or high light for 3 d. Total proteins were extracted from the peels and were incubated with purified MdTCP46-GST protein for 0–6 h. For treatment with the proteasome inhibitor MG132, total proteins of fruit subjected to low light were pre-treated with 100 µM MG132 for 0.5 h before the sampling began. ACTIN was used as an internal reference. (D) Apple peel injection assays. Uncolored ‘Red Delicious’ fruit were injected with mixed vectors or *A. tumefaciens* solutions and stored in a phytotron under the high-light treatment for 3 d. pIR, IL60-1+IL60-2; MdTCP46-pIR, IL60-1+MdTCP46-IL60-2; TRV, TRV1+ TRV2; MdTCP46-TRV, TRV1+MdTCP46-TRV2. pIR are overexpressors (OX); TRV are antisense suppressors (Anti). (E) Anthocyanin contents of the fruit peels shown in (D). Contents are expressed relative to pIR, the value of which was set as 1. (F) Expression of genes related to anthocyanin biosynthesis in the fruit peels shown in (D) as determined by qRT-PCR. Expression was normalized to the *actin* gene. Values are expressed relative to pIR, which were set as 1. (G) qRT-PCR detection of *MdTCP46* expression levels in Arabidopsis Col-0 seedlings and in overexpressing transgenic lines. The lower panels show the *ACTIN* gene, which was used as the internal control. (H) Phenotypes of Arabidopsis Col-0 and *MdTCP46*-overexpressing lines and (I) relative expression of *MdTCP46*. Expression was normalized to the *ACTIN* gene. Values are expressed relative to Col-0, which was set as 1. All experiments were performed three times with similar results, and representative data from one experiment are shown. Data are means (±SD), *n*=6. Different letters indicate significant differences as determined by one-way ANOVA and LSD tests (*P*<0.05).

MdTCP46 is a TCP family protein and was found to contain a conserved bHLH domain at the N terminus (Fig. 2A). Compared to TCP proteins from different orspeciesganisms, it exhibited the highest homology with the *Pyrus bretschneideri* protein, PbTCP.

When apple fruit were stored under different light intensities, qRT-PCR analysis indicated that moderate and high light significantly induced the expression of *MdTCP46* (Fig. 2B). The abundance of the MdTCP46 protein was also examined. MdTCP46-GST, an *E. coli* fusion protein, was probed with GST antibodies under either low or high light treatment. The results indicated that high light delayed MdTCP46 protein degradation (Fig. 2C). Furthermore, inclusion of the MG132 proteasome inhibitor blocked degradation completely, indicating that MdTCP46 is degraded by the 26S-proteasome machinery. These results suggested that *MdTCP46* was responsive to high light intensity at both the transcriptional and post-translational levels.

To assess the biological role of MdTCP46 in the regulation of anthocyanin biosynthesis, transgenic apple fruit and Arabidopsis were generated (Supplementary Fig. S2B). The MdTCP46-pIR construct was used to generate *MdTCP46*-overexpression (-OX) lines and the MdTCP46-TRV construct was used to generate *MdTCP46*-antisense (-Anti) lines. Compared to the WT controls, overexpression of *MdTCP46* in both apple fruit and Arabidopsis seedlings clearly increased anthocyanin accumulation whilst antisense suppression decreased it (Fig. 2D–I). These results indicate an essential and positive role of *MdTCP46* in anthocyanin accumulation.

### MdTCP46 is essential for anthocyanin biosynthesis mediated by high light intensity

**Fig. 3.**
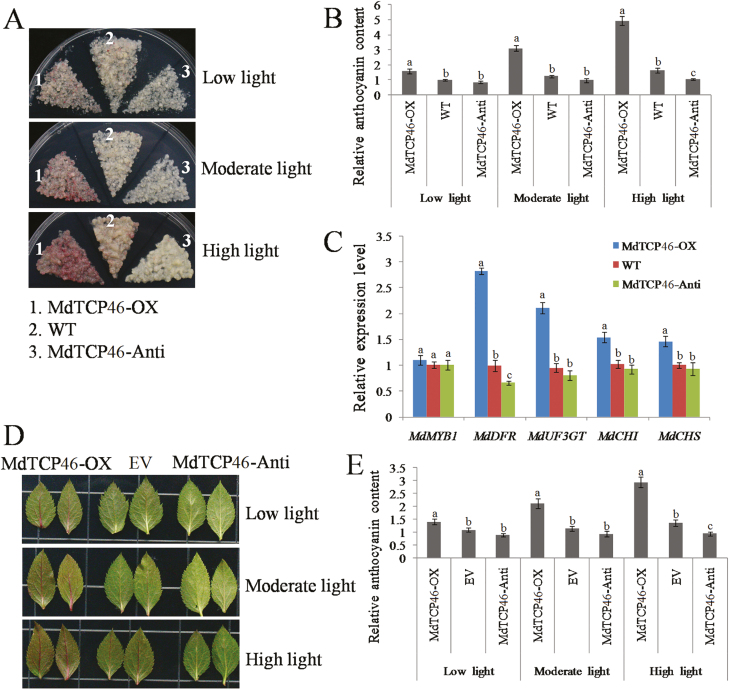
MdTCP46 promotes light-induced anthocyanin biosynthesis in apple. Transgenic apple calli (15 d old) and leaves of *MdTCP46*-overexpressing (-OX) and *MdTCP46*-antisense (-Anti) were subjected to different light intensities for 5 d. (A) Phenotypes of calli of the transgenic lines and the wild-type (WT) and (B) their anthocyanin contents. The content of low light-treated WT was used as a reference and set to 1. (C) The expression of genes related to anthocyanin biosynthesis in apple calli as determined by qRT-PCR. The expression of the WT was used as a reference and set to 1. (D) Phenotypes of transient transgenic leaves the empty-vector control (EV) and (E) their anthocyanin contents. The content of low light-treated EV was used as a reference and set to 1. All experiments were performed three times with similar results, and representative data from one experiment are shown. Data are means (±SD), *n*=4. Different letters indicate significant differences as determined by one-way ANOVA and LSD tests (*P*<0.05).

We generated stable transgenic apple calli and transient transgenic leaves with either overexpression of *MdTCP46* (*MdTCP46*-OX) or with antisense suppression of *MdTCP46* (*MdTCP46*-Anti) (Supplementary Fig. S2C, D).Compared to the WT controls, overexpression of *MdTCP46* promoted anthocyanin accumulation in both the calli and leaves, whilst antisense suppression of *MdTCP46* reduced it (Fig. 3). The expression of five genes related to anthocyanin biosynthesis were examined in the calli, namely *MdMYB1*, *MdDFR*, *MdUF3GT*, *MdCHI*, and *MdCHS*. All five were expressed at higher levels when *MdTCP46* was overexpressed (Fig. 3C). The effect of *MdTCP46* expression on anthocyanin accumulation was more obvious as light intensity increased (Fig. 3A, B, D, E), indicating that it was essential for accumulation mediated by high light intensity.

### MdTCP46 promotes anthocyanin biosynthesis when *MdMYB1* is suppressed

**Fig. 4.**
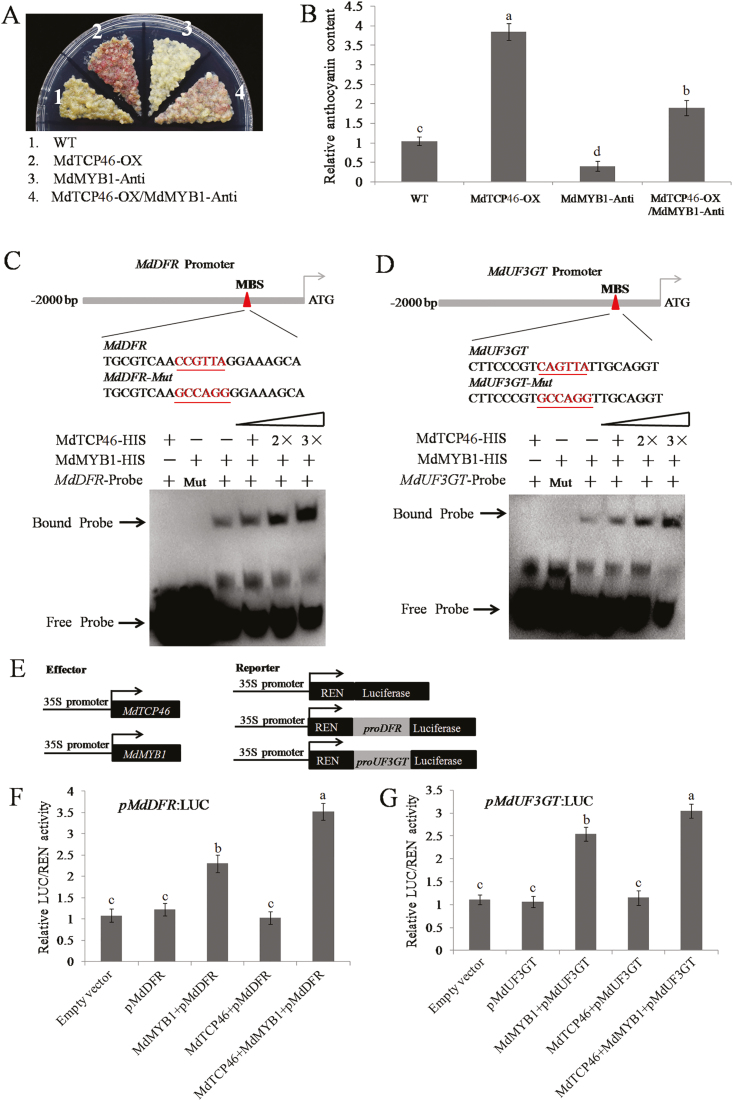
MdTCP46 enhances the transcriptional activity of MdMYB1 on *MdDFR* and *MdUF3GT*. (A, B) Apple calli (15 d old) of the wild-type (WT), *MdTCP46*-overexpression (-OX), *MdMYB1*-antisense (-Anti), and *MdTCP46*-OX/*MdMYB1*-Anti (overexpression of *MdTCP46* in the background of *MdMYB1*-Anti) were subjected to high light for 5 d. (A) Phenotypes and (B) anthocyanin levels. The content of the WT was used as a reference and set to 1. (C, D) Electromobility shift assays using *MdDFR* and *MdUF3GT* promoter probes. The MBS sequences for MdMYB1 binding and their equivalent mutant forms (-Mut) are underlined. + indicates the presence of corresponding proteins or probes, and − indicates the absence of corresponding of proteins. 2× and 3× indicate increased protein contents. (E) Schematic representation of the LUC reporter vector containing the *MdDFR* and *MdUF3GT* promoters and effector vectors expressing *MdTCP46* or *MdMYB1* under the control of the 35S promoters. The ORFs of *MdTCP46* and *MdMYB1* were fused to the pGreenII 62-SK vector. The promoter sequences of *MdDFR* and *MdUF3GT* were cloned into the pGreenII 0800-LUC vector. (F, G) LUC/REN activities detected by the reporter systems described in (E), which tested the effects of MdMYB1, MdTCP46, and MdMYB1+MdTCP46 on the expression of (F) *MdDFR* and (G) *MdUF3GT.* Empty vector, pGreenII 62-SK + pGreenII 0800-LUC; pMdDFR, pGreenII 62-SK + *proMdDFR-*pGreenII 0800-LUC; pMdUF3GT, pGreenII 62-SK + *proMdUF3GT*-pGreenII 0800-LUC; MdMYB1+pMdDFR, MdMYB1-pGreenII 62-SK + *proMdDFR*-pGreenII 0800-LUC; MdMYB1+pMdUF3GT, MdMYB1-pGreenII 62-SK + *proMdUF3GT*-pGreenII 0800-LUC; MdTCP46+pMdDFR, MdTCP46-pGreenII 62-SK + *proMdDFR*-pGreenII 0800-LUC; MdTCP46+pMdUF3GT, MdTCP46-pGreenII 62-SK + *proMdUF3GT*-pGreenII 0800-LUC; MdTCP46+MdMYB1+pMdDFR, MdTCP46-pGreenII 62-SK + MdMYB1-pGreenII 62-SK + *proMdDFR*-pGreenII 0800-LUC; MdTCP46+MdMYB1+pMdUF3GT, MdTCP46-pGreenII 62-SK + MdMYB1-pGreenII 62-SK + *proMdUF3GT*-pGreenII 0800-LUC. LUC/REN activities of the empty vector were used as references and set to 1. All experiments were performed three times with similar results, and representative data from one experiment are shown. Data are means (±SD), *n*=3. Different letters indicate significant differences as determined by one-way ANOVA and LSD tests (*P*<0.05).

Since both MdTCP46 and MdMYB1 play positive roles in anthocyanin biosynthesis induced by high light intensity in apple, we examined the functional relationship between them. Transgenic apple calli were generated to overexpress *MdTCP46* in a *MdMYB1*-suppression background (i.e. *MdTCP46*-OX/*MdMYB1*-Anti) (Supplementary Fig. S2A). Control samples expressing the *MdTCP46*-OX or *MdMYB1*-Anti constructs individually showed increased and decreased anthocyanin accumulation, respectively (Fig. 4A, B), whilst samples expressing both the constructs showed increased accumulation, but to a lesser extent than observed in *MdTCP46*-OX. These results indicated that MdTCP46 promoted anthocyanin biosynthesis when *MdMYB1* was suppressed.

### MdTCP46 enhances the binding activity of MdMYB1 to its target gene promoters

To determine the functions of MdTCP46 and MdMYB1 during anthocyanin biosynthesis, we examined their roles in binding to the promoter sequences of target genes. First, EMSAs were developed using the MdTCP46-HIS and MdMYB1-HIS fusion proteins and the biotin-labeled probes of *MdDFR* and *MdUF3GT* (Fig. 4C, D; Supplementary Tables S1, S2). Mutated forms of the *MdDFR-Mut* and *MdUF3GT-Mut* probes were used as negative controls. The results showed that MdMYB1-HIS alone was able to bind to the MdDFR-Probe and MdUF3GT-Probe, which was consistent with previous studies (Takos *et al.*, 2006; Ban *et al.*, 2007; Espley *et al.*, 2007). However, the binding became stronger in the presence of MdTCP46-HIS fusion proteins (Fig. 4C, D), indicating that MdTCP46 could improve the binding activity of MdMYB1 to the *MdDFR* and *MdUF3GT* promoters.

Dual-luciferase assays were also conducted to support the EMSA observations. *MdTCP46* and *MdMYB1* were expressed separately under the control of 35S promoters on the pGreenII 62-SK plasmid vector as effectors, and *LUC* was used as a reporter behind the *MdDFR* or *MdUF3GT* promoter in the pGreenII 0800-LUC plasmid vector (Fig. 4E). It was clear that MdMYB1 alone was able to drive the expression of the luciferase gene, while MdTCP46 could not (Fig. 4F, G). *LUC* expression was increased significantly when the constructs expressing both proteins were present, suggesting that MdTCP46 enhanced the binding activity of MdMYB1 to the *MdDFR* and *MdUF3GT* promoters.

### MdBT2 physically interacts with MdTCP46

**Fig. 5.**
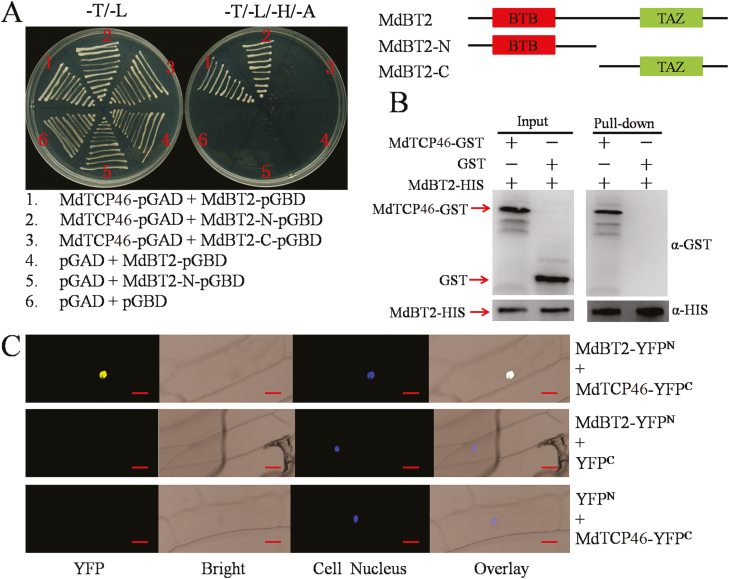
MdBT2 interacts with MdTCP46. (A) Yeast two-hybrid assays. The ORF of *MdTCP46* and the indicated regions of *MdBT2* (i.e. *MdBT2*, *MdBT2* N terminal, and *MdBT2* C terminal; see schematic diagram to right) were fused to the pGAD and pGBD vectors. The pGAD and pGBD empty vectors were used as negative controls. –T/–L, SD medium lacking Trp and Leu; –T/–L/–H/–A, SD medium lacking Trp, Leu, His, and Ade. (B) Pull-down assays. The ORFs of *MdBT2* and *MdTCP46* were fused to the pET32a and pGEX4T-1 vectors, respectively. The MdBT2-HIS and MdTCP46-GST fusion proteins were obtained from *E. coli* expressions. GST proteins were used as negative controls. The eluted solution was probed with HIS and GST antibodies. (C) Bimolecular fluorescence complementation assays. The ORFs of *MdBT2* and *MdTCP46* were fused to the YFP^N^ and YFP^C^ vectors, respectively. Fluorescence was examined using a confocal laser-scanning microscope. Scale bars are 10 μm.

We next attempted to identify the potential protein interacting partners of MdTCP46. The bric-a-brac/tramtrack/broad (BTB) family protein MdBT2 has been identified and widely investigated as a repressor of anthocyanin accumulation (An *et al.*, 2018*a*, 2018*d*, 2019*a*, 2019*c*, 2020; Wang *et al.*, 2018). We therefore conducted three assays to verify the physical interaction between the MdTCP46 and MdBT2 proteins. First, *MdTCP46* was cloned into the pGAD424 vector. *MdBT2*, its N-terminus (*MdBT2*-N), and C-terminus (*MdBT2*-C) were individually cloned into the pGBT9 vector (Fig. 5A). The results of Y2H assays revealed that only yeast cells co-transformed with both the full-lengths of *MdTCP46* and *MdBT2* survived on selective medium (Fig. 5A), indicating a direct interaction between MdTCP46 and MdBT2 in the cells. Second, pull-down assays were performed that utilized the *E. coli* expression of MdBT2-HIS and MdTCP46-GST fusion proteins (Fig. 5B). The polyhistidine binding resin pulled-down the MdBT2-HIS and MdTCP46-GST fusion proteins, but not when the GST tag was present alone (Fig. 5B). Thus, heterologously expressed MdTCP46 and MdBT2 proteins could interact *in vitro*. Third, BiFC assays were performed using onion epidermal cells, and the expression system was transformed with *MdTCP46* linked to the C-terminus of YFP (*MdTCP46*-YFP^C^), *MdBT2* linked to the N-terminus of YFP (*MdBT2*-YFP^N^), or both. The results showed that MdTCP46-YFP^C^ interacted with MdBT2-YFP^N^ in the nucleus (Fig. 5C). Collectively, these results suggested that MdTCP46 interacts with MdBT2 directly *in vivo* and *in vitro*.

### MdBT2 is a negative regulator in anthocyanin biosynthesis induced by high light

**Fig. 6.**
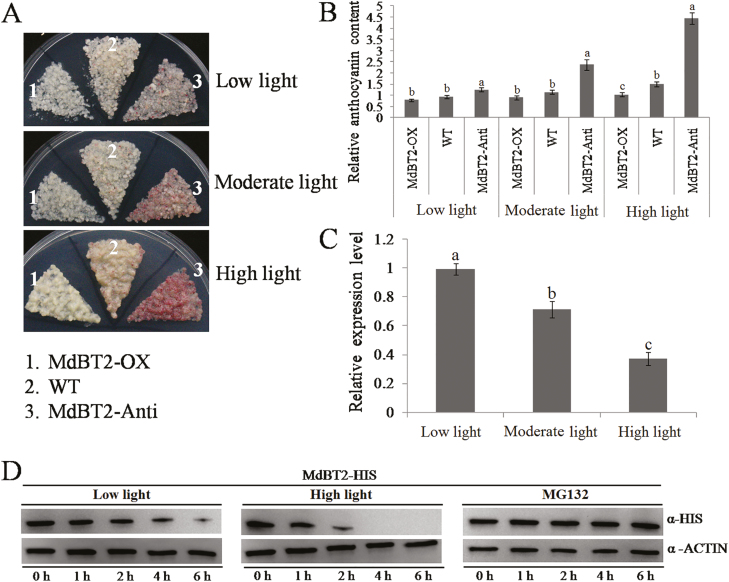
MdBT2 negatively regulates light-induced anthocyanin biosynthesis. Apple calli (15 d old) of the wild-type (WT) and transgenic *MdBT2*-overexpressing (-OX) and *MdBT2*-antisense (-Anti) lines were subjected to different light intensities for 5 d. (A) Phenotypes and (B) anthocyanin levels. The anthocyanin content the WT under the low-light treatment was used as a reference and set to 1. (C) Expression of *MdBT2* in response to the different light intensities as determined using qRT-PCR. Expression was normalized to the *ACTIN* gene. Values are expressed relative to the low-light treatment, which was set to 1. (D) Detection of the MdBT2-HIS fusion protein after low-and high-light treatments. ‘Red Delicious’ fruit were subjected to the light treatments for 3 d. The total proteins extracted from the peels were incubated with purified MdBT2-HIS protein for 0–6 h. For the treatment with the proteasome inhibitor MG132, the total proteins fruit subjected to low light were pre-treated with 100 µM MG132 for 0.5 h before the sampling began. ACTIN was used as an internal reference. All experiments were performed three times with similar results, and representative data from one experiment are shown. Data are means (±SD), *n*=3. Different letters indicate significant differences as determined by one-way ANOVA and LSD tests (*P*<0.05).

MdBT2 has previously been identified to repress anthocyanin biosynthesis (An *et al.*, 2018*a*; Wang *et al.*, 2018). Here, we specifically examined the involvement of MdBT2 in anthocyanin biosynthesis mediated by different light intensities in apple. *MdBT2*-OX and *MdBT2*-Anti calli were generated (Supplementary Fig. S2E). The overexpression of *MdBT2* reduced the accumulation of anthocyanin under high light, while suppression of *MdBT2* increased anthocyanin levels under all the treatments but particularly under high light (Fig. 6A, B). These results confirmed that MdBT2 functions as a negative regulator of anthocyanin biosynthesis in apple and is especially important under high light conditions. Interestingly, the expression of *MdBT2* seemed to be regulated by the different light intensities, and it was noticeably downregulated under the high intensity treatment (Fig. 6C). *In vitro* protein degradation assays using *E. coli* expressed MdBT2-HIS fusion proteins indicated that the high-light treatment accelerated the degradation of the MdBT2 protein (Fig. 6D), and this appeared to be mediated by 26S proteasome as the presence of the MG132 proteasome inhibitor completely abolished the degradation of the MdBT2-HIS fusion protein. Thus, high light clearly suppressed the expression of *MdBT2* at both the transcriptional and post-translational levels.

### MdBT2 suppresses the role of MdTCP46 in anthocyanin biosynthesis induced by high light by inducing its degradation

**Fig. 7.**
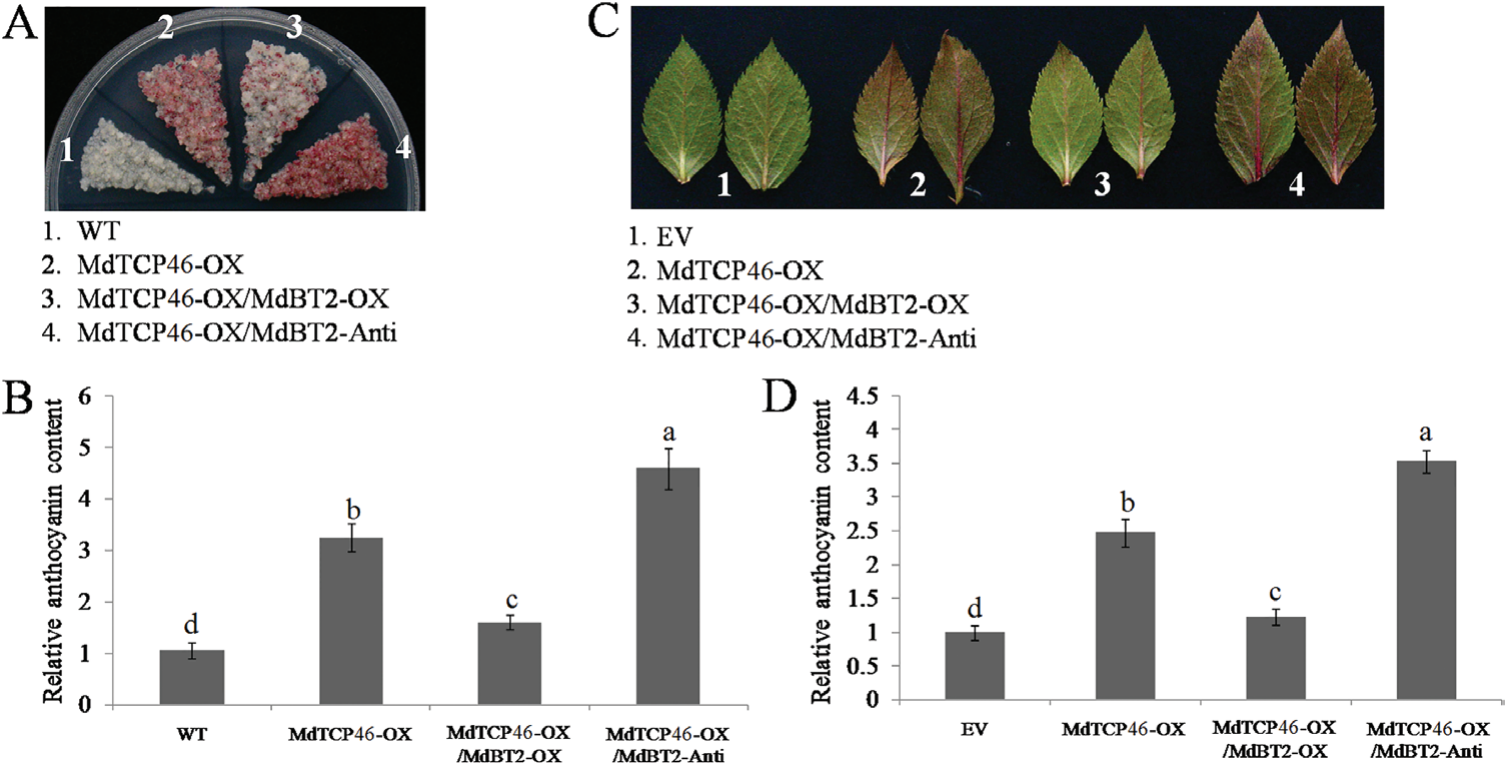
MdBT2 negatively regulates MdTCP46-promoted anthocyanin biosynthesis. Transgenic apple calli (15 d old) and leaves of *MdTCP46*-overexpressing (-OX), *MdTCP46*-OX/*MdBT2*-OX (overexpression of *MdBT2* in the background of *MdTCP46*-overexpression), and *MdTCP46*-OX/*MdBT2*-Anti (antisense suppression of *MdBT2* in the background of *MdTCP46*-overexpression were subjected to high light intensity for 5 d. (A) Phenotypes of calli of the transgenic lines and the wild-type (WT) and (B) their anthocyanin contents. The content of the WT was used as a reference and was set to 1. (C) Phenotypes of transient transgenic leaves and the empty vector (EV) and (D) their anthocyanin contents. The content of the EV was used as a reference and was set to 1. All experiments were performed three times with similar results, and representative data from one experiment are shown. Data are means (±SD), *n*=4. Different letters indicate significant differences as determined by one-way ANOVA and LSD tests (*P*<0.05).

Given that MdTCP46 played a positive role and its interacting partner MdBT*2* played a negative role in anthocyanin accumulation induced by high light (Figs 3, 6A, B), we examined the physiological relationship between these two genes. *MdTCP46*-OX was co-expressed with *MdBT2*-OX or *MdBT2*-Anti in apple calli and leaves under high light (Supplementary Fig. S2F–H). Compared with the control, all the transgenic plants showed significant increases in anthocyanin content in both calli and leaves (Fig. 7). The greatest increases were observed when expression of *MdBT2* was suppressed, whilst overexpression of *MdBT2* and *MdTCP46* together resulted in anthocyanin levels that were lower than when *MdTCP46* was overexpressed alone (Fig. 7B, D). These results suggested that *MdTCP46* plays a positive role whilst *MdBT2* plays a negative role in anthocyanin accumulation under high-light conditions. In addition, *MdBT2* suppressed the positive role played by *MdTCP46*.

**Fig. 8.**
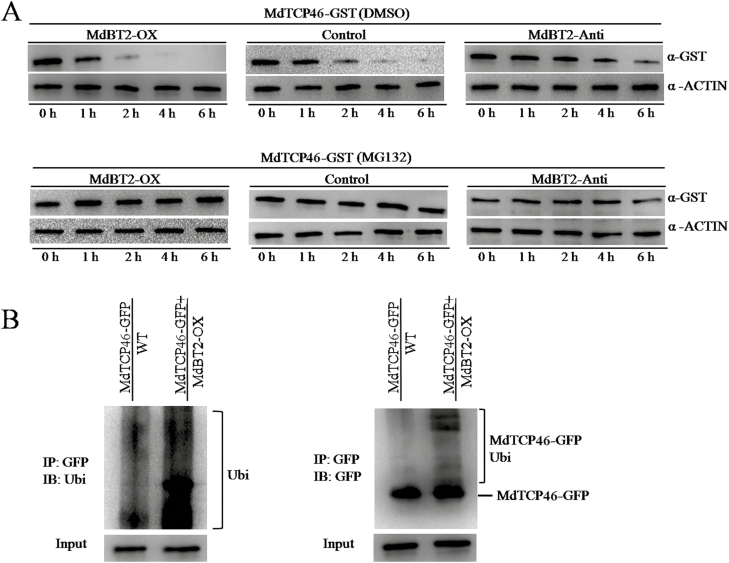
MdBT2 degrades the MdTCP46 protein. (A) Detection of MdTCP46-GST fusion proteins in stability assays. Protein extracts were taken from 15-d-old calli grown under dark conditions for the wild-type (WT) and transgenic lines of *MdBT2*-overexpression (-OX) and MdBT2-antisense (-Anti) and were treated for 0.5 h with either 100 µM of the proteasome inhibitor MG132 dissolved in DMSO or in DMSO alone (blank control), before being incubated with MdTCP46-GST protein for 0–6 h. ACTIN was used as an internal reference. (B) MdBT2 promotes the ubiquitination of the MdTCP46 protein *in vivo*. MdTCP46-GFP was immunoprecipitated using the GFP antibody from the two transgenic calli *MdTCP46*-GFP and *MdTCP46*-GFP/*MdBT2*-OX. The immunoprecipitated proteins were examined using antibodies for ubiquitin (left) and GFP (right).

To further explore the interaction mechanism between MdTCP46 and MdBT2, *in vitro* protein degradation assays were conducted. The MdTCP46-GST fusion protein was incubated with total protein extracts from *MdBT2*-OX and *MdBT2*-Anti calli. Consistent with the patterns of anthocyanin accumulation (Fig. 7A, B), degradation of the MdTCP46-GST fusion protein was facilitated by *MdBT2* overexpression, but delayed by *MdBT2* suppression (Fig. 8A). This effect was abolished by the presence of MG132, indicating that the protein degradation was through the 26S proteasome pathway. Indeed, when the MdTCP46-GFP fusion protein was incubated with protein extracts from *MdBT2*-OX calli, strong ubiquitination patterns were observed using Ubi and GFP antibodies with various forms of ubiquitinated MdTCP46-GFP proteins being detected (Fig. 8B), indicating that a considerable amount of the fusion proteins had been partially degraded and lost the GFP tag. This was consistent with the fact that MdBT2 suppressed the function of MdTCP46 by facilitating its degradation through the 26S proteasome pathway.

### MdTCP46 specifically interacts with the MdMYB1 and MdBT2 proteins

There are several TCP family proteins, some of which may have similar or opposing regulatory functions in the same signaling pathways (Li and Zachgo, 2013; Viola *et al.*, 2016). When we screened the MdMYB1-interacting proteins, MdTCP3, MdTCP12, and MdTCP21 were screened out (Xu *et al.*, 2014). Therefore, we cloned these three TCP proteins. In contrast to MdTCP46, none of them exhibited direct interactions with MdMYB1 and MdBT2 in the Y2H assays (Supplementary Fig. S4). Hence, MdTCP46 seems to specifically interact with the MdMYB1 and MdBT2 proteins.

## Discussion

Anthocyanin biosynthesis is regulated by various environmental stimuli (Winkel-Shirley, 2001, 2002; Carbone *et al.*, 2009; Jaakola, 2013; Honda and Moriya, 2018), and light, especially at high intensity, has been implicated in promoting biosynthesis in many species including apple (Jaakola, 2013; Trojak and Skowron, 2017; Zhang *et al.*, 2018*b*). A positive correlation exists between anthocyanin accumulation and light intensity (Jaakola, 2013; Trojak and Skowron, 2017; Zhang *et al.*, 2018*b*). Although many proteins have been shown to participate synergistically in light-modulated anthocyanin accumulation. including MYB, BBX, ERF, and HY5 (Ubi *et al.*, 2006; Gong *et al.*, 2015; Henry-Kirk *et al.*, 2018; Zhang *et al.*, 2018*a*; An *et al.*, 2019*a*; Fang *et al.*, 2019; Ni *et al.*, 2019), the underlying molecular mechanisms are not fully understood. Here, we found that MdTCP46 and MdMYB1 play positive roles in anthocyanin biosynthesis induced by high light. In addition, the BTB-domain protein MdBT2 acted as a repressor of anthocyanin biosynthesis under different light intensities by regulating the stability of the MdTCP46 protein.

MdMYB1 is known to be responsive to light treatment in apple (Takos *et al.*, 2006; Ban *et al.*, 2007; Espley *et al.*, 2007). The importance of MdMYB1 in light-induced anthocyanin biosynthesis has been well established, and it functions by directly activating the expression of genes related to biosynthesis, such as *DFR* and *UF3GT* (Takos *et al.*, 2006; Ban *et al.*, 2007; Espley *et al.*, 2007; An *et al.*, 2018*d*, *c*2019*c*, 2020). In our current study, the promotion of anthocyanin accumulation by different light intensities was confirmed in apple fruit and calli (Supplementary Fig. S1). MdMYB1 was shown to play a key role, as suppression of *MdMYB1* resulted in a significant decrease in the accumulation of anthocyanin in apple calli under different light intensities.

In Arabidopsis, overexpression of *TCP15* decreases anthocyanin biosynthesis and high light inactivates the TCP15 protein (Viola *et al.*, 2016), whilst TCP3 plays a positive role in the regulation of biosynthesis by interacting with MYB proteins (Li and Zachgo, 2013). Here, we demonstrated that MdTCP46 interacted with MdMYB1 in Y2H, pull-down, and BiFC assays (Fig. 1).

**Fig. 9.**
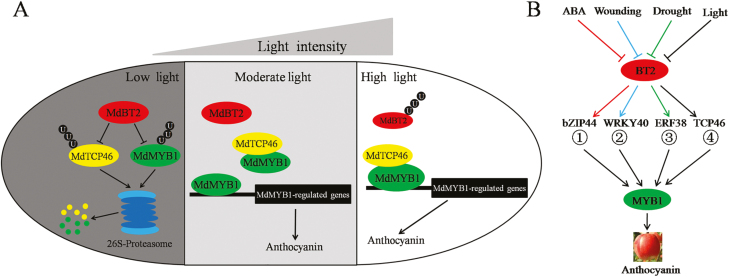
Proposed model of the functioning of MdTCP46 in light-induced anthocyanin biosynthesis. (A) MdTCP46 interacts with MdMYB1 to increase its transcriptional activity and to enhance its binding to target genes, thereby promoting light-mediated anthocyanin biosynthesis. Under low light, MdBT2 interacts with MdTCP46 to ubiquitinate and degrade it, thus negatively regulating MdTCP46-promoted anthocyanin biosynthesis. Under high light, the transcription of *MdTCP46* is up-regulated and anthocyanin biosynthesis is stimulated. Light inhibits *MdBT2* expression, which in turn releases the MdBT2-enhanced degradation of the MdTCP46 protein, thereby contributing to light-induced anthocyanin biosynthesis. (B) The central role of MdBT2 in anthocyanin biosynthesis mediated by multiple stresses. (1) An *et al.* (2018*d*); (2) An *et al.* (2019*c*); (3) An *et al.* (2020); (4) present study.

The transcription of *MdTCP46* was stimulated as light intensity increased (Fig. 2B), and the high light treatment dramatically increased the stability of MdTCP46 (Fig. 2C). This indicates that high light intensity affects the expression of *MdTCP46* at both the transcriptional and post-transcriptional levels. We found that MdTCP46 positively regulated anthocyanin accumulation in response to different light intensities by up-regulating the expression of biosynthesis genes (Fig. 2D–I; Fig. 3). Overexpression of *MdTCP46* did not interfere with *MdMYB1* expression (Fig. 2F, 3C), which prompted us to examine whether MdTCP46 affected the binding activity of MdMYB1 to its target genes. Previous reports have indicated that MdMYB1 is able to bind to the promoter fragments of *DFR* and *UF3GT* (Takos *et al.*, 2006; Ban *et al.*, 2007; Espley *et al.*, 2007; An *et al.*, 2018*d*, 2019*c*, 2020). As expected, we found that interaction with MdTCP46 did indeed facilitate the binding activity of MdMYB1 to its target genes (Fig. 4C–G), providing evidence that this interaction contributes to anthocyanin biosynthesis induced by high light. Combining our results with those of previous studies, it appears that MdMYB1 integrates the accumulation of anthocyanin in response to multiple stresses by interacting with different hormones and environmental signal-response factors, such as the ABA-response factor MdbZIP44, the wounding-response factor MdWRKY40, the drought-response factor MdERF38, and the light-response factor MdTCP46 (Takos *et al.*, 2006; Ban *et al.*, 2007; Espley *et al.*, 2007; An *et al.*, 2018*d*, 2019*c*, 2020; Fig. 9B), in each of which MdMYB1 plays a core regulatory role. Therefore, it seems likely that there may be functional redundancy in these different response factors.

In Arabidopsis, AtTCP15 is a repressor of anthocyanin biosynthesis and may act by inducing the expression of a PAP1 repressor and other anthocyanin regulatory genes (Viola *et al.*, 2016). Although MdTCP46 is a TCP15-like protein, it exhibits a different function to AtTCP15 as it is a positive regulator of anthocyanin biosynthesis and is involved in regulating synthesis by interacting with the MBW complex (Viola *et al.*, 2016). Thus species differences in function may exist.

To date, the transcriptional and post-transcriptional regulation of TCP proteins have rarely been investigated. Several reports have showed that TCP proteins may be regulated by miR319 (Schommer *et al.*, 2014; Bresso *et al.*, 2018; Palatnik and Weigel, 2019, Preprint). Since our high light intensity treatment improved the stability of the MdTCP46 protein (Fig. 2C), we examined interacting partners that might have potential roles in regulating this stability. The BTB protein MdBT2 was found to interact with MdTCP46 (Fig. 5). The expression of *MdBT2* was inhibited as light intensity increased at both the transcriptional and post-transcriptional levels (Fig. 6C, D), which contrasted with the response pattern of MdTCP46 (Fig. 2B, C). We found that MdBT2 negatively regulated the biosynthesis of anthocyanin under high light intensity by degrading the MdTCP46 protein directly (Figs 7, 8). Based on these findings, we propose that MdBT2 mainly functions under low light conditions while high light intensity triggers the accumulation of MdTCP46, which is probably due to the degradation of MdBT2 that is promoted by high light.

A proposed model that summarizes the functioning of MdTCP46 in light-induced anthocyanin biosynthesis is shown in Fig. 9A. MdTCP46 improves the binding activity of MdMYB1 to the promoters of *MdDFR* and *MdUF3GT* by directly interacting with it, and thereby promotes the biosynthesis of anthocyanin that is induced by high light. Under low light intensity, MdBT2 ubiquitinates and degrades the MdTCP46 and MdMYB1 proteins, thus decreasing MdTCP46-promoted accumulation of anthocyanin. As light intensity increases, the expression of *MdBT2* is inhibited while that of *MdTCP46* is activated, thereby triggering high light intensity-induced anthocyanin biosynthesis. Thus, we speculate that MdBT2 may act as a dose-responder of light intensity. Our discovery of this ‘MdBT2–MdTCP46’ ubiquitination regulation module provides new insights that should aid future studies on the post-transcriptional regulation of TCP proteins.

BT2 is a key component of the BT2-CUL3-RBX1 ubiquitin ligase complex, and multiple proteins have been confirmed to be targets for ubiquitination by BT2 (Figueroa *et al.*, 2005; Mandadi *et al.*, 2009; Zhao *et al.*, 2016; An *et al.*, 2018*a*; Wang *et al.*, 2018). In addition to MdTCP46, different types of MdBT2-interacting proteins have been identified, including MdMYB1, MdMYB9, MdMYB23, MdbHLH93, MdbHLH104, MdBBX22, MdbZIP44, MdWRKY40, and MdERF38 (Zhao *et al.*, 2016; An *et al.*, 2018*a*, 2018*b*, 2018*d*, 2019*a*, 2019*b*, 2019*c*, 2020; Wang *et al.*, 2018). MdBT2 regulates the stability of these proteins through direct interactions and participates in multiple stress-response processes, suggesting that it may be a multifunctional fine-tuning protein that integrates a post-transcriptional regulatory network in response to multiple stress signals. The observations that different transcription factors function as the interaction proteins of both MdBT2 and MdMYB1 indicate that the ‘BT2-interacting proteins–MYB1’ module may be a central component of anthocyanin biosynthesis induced by multiple stresses (Fig. 9B).

## Supplementary data

Supplementary data are available at *JXB* online.

Fig. S1. Mediation of anthocyanin biosynthesis at different light intensities is dependent on MdMYB1.

Fig. S2. Identification of transgenic plant material by qRT-PCR.

Fig. S3. Protein sequence alignment of TCP proteins in apple and Arabidopsis.

Fig. S4. Yeast two-hybrid assays showing that MdTCP46 specifically interacts with MdMYB1 and MdBT2.

Table S1. The promoter sequence of *MdDFR*.

Table S2. The promoter sequence of *MdUF3GT*.

Table S3. Primers used for gene expression analysis and vector construction.

eraa056_suppl_Supplementary_Figues_S1_S4

eraa056_suppl_Supplementary_Tables_S1_S3
