# Supplementary material for: Dynamic regulation of anthocyanin biosynthesis at different light intensities by the BT2-TCP46-MYB1 module in apple
Source: J Exp Bot. 2020 Jan 30;71(10):3094–109. doi: 10.1093/jxb/eraa056 (PMC7475178; doi:10.1093/jxb/eraa056)

Supplemental Figures

Supplemental Figure 1. Different light intensity-mediated anthocyanin biosynthesis is dependent on MdMYB1.

(A) Appearances of apple fruits. (B) Anthocyanin contents. Uncolored ‘Red Delicious’ apple fruits were treated for 6 days with dark, low light, moderate light, and high light, and anthocyanin content was measured. Anthocyanin content of apple fruits with low light treatment was used as the reference and set to 1. Each experiment contained 3-4 apple fruits per treatment. (C) The expressions of anthocyanin biosynthesis related genes *MdMYB1*, *MdDFR*, *MdUF3GT*, *MdCHI*, and *MdCHS* were examined by qRT-PCR. The expressions detected in the apple fruits treated with low light were used as the references and set to 1. (D) Phenotypes and (E) anthocyanin levels of apple calli in the dark or different light intensity treatments (low light, moderate light, and high light). WT: wild-type; MdMYB1-Anti: *MdMYB1* antisense suppression. Apple calli of 15-day-old were treated with dark or different light intensity treatments for 10 days. Anthocyanin content of low light-treated wild-type apple calli were used as the reference and set to 1. All experiments were performed three times with similar results, and representative data from one repetition were shown. Error bars denoted standard deviation. Different letters above the bars indicated significant difference ($P < 0.05$) as obtained by one-way ANOVA and LSD test.

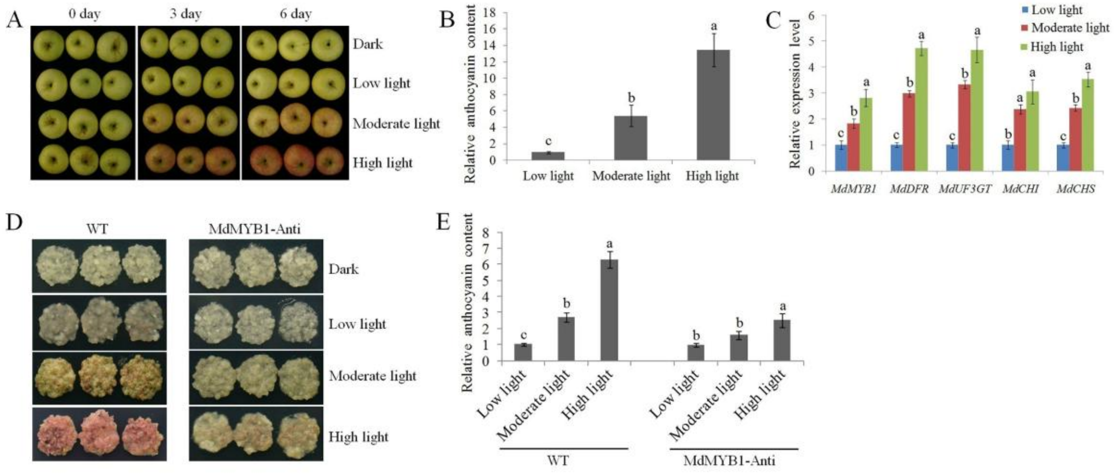

Supplemental Figure 2. Identification of transgenic plant materials by qRT-PCR.
(A) The expression of *MdMYB1* in apple calli. WT: wild-type; MdMYB1-Anti: *MdMYB1* antisense suppression. MdTCP46-OX/MdMYB1-Anti: overexpression of *MdTCP46* in the background of MdMYB1-Anti. The value for WT was set to 1. (B) The expression of *MdTCP46* in fruit peels around the injection sites. The value for pIR was set to 1. (C) The expression of *MdTCP46* in apple calli. WT: wild-type; MdTCP46-OX: *MdTCP46*-overexpression; MdTCP46-Anti: *MdTCP46* antisense suppression. The value for WT was set to 1. (D) The expression of *MdTCP46* in transient transgenic apple leaves. EV: empty vector; MdTCP46-OX: *MdTCP46*-overexpression; MdTCP46-Anti: *MdTCP46* antisense suppression. The value for EV was set to 1. (E) The expression of *MdBT2* in apple calli. WT: wild-type; MdBT2-OX: *MdBT2*-overexpression; MdBT2-Anti: *MdBT2* antisense suppression. The value for WT was set to 1. (F) The expression of *MdBT2* in *MdTCP46-MdBT2* transgenic apple calli. WT: wild-type; MdTCP46-OX/MdBT2-OX: overexpression of *MdBT2* in the background of *MdTCP46*-overexpression; MdTCP46-OX/MdBT2-Anti: antisense suppression of *MdBT2* in the background of *MdTCP46*-overexpression. The value for MdTCP46-OX was set to 1. (G) The expression of *MdTCP46* in *MdTCP46-MdBT2* transgenic apple leaves. EV: empty vector; MdTCP46-OX: *MdTCP46*-overexpression; MdTCP46-OX/MdBT2-OX: overexpression of *MdBT2* in the background of *MdTCP46*-overexpression; MdTCP46-OX/MdBT2-Anti: antisense suppression of *MdBT2* in the background of *MdTCP46*-overexpression. The value for EV was set to 1. (H) The expression of *MdBT2* in *MdTCP46-MdBT2* transgenic apple leaves. MdTCP46-OX: *MdTCP46*-overexpression; MdTCP46-OX/MdBT2-OX: overexpression of *MdBT2* in the background of *MdTCP46*-overexpression; MdTCP46-OX/MdBT2-Anti: antisense suppression of *MdBT2* in the background of *MdTCP46*-overexpression. The value for MdTCP46-OX was set to 1.

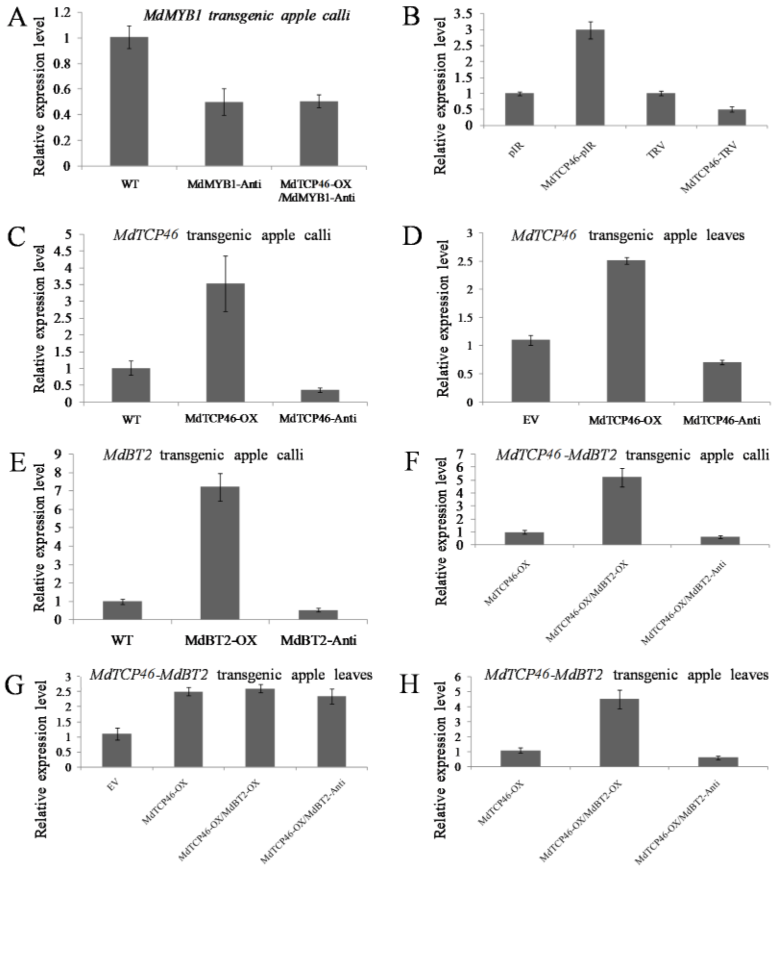

Supplemental Figure 3. Protein sequence alignment of TCP proteins.
(A) NCBI showed the MdMYB1 interaction protein was a TCP15-like protein. (B) Protein sequence alignment of MdTCP46 and AtTCP3. (C) Protein sequence alignment of MdTCP46 and AtTCP15.

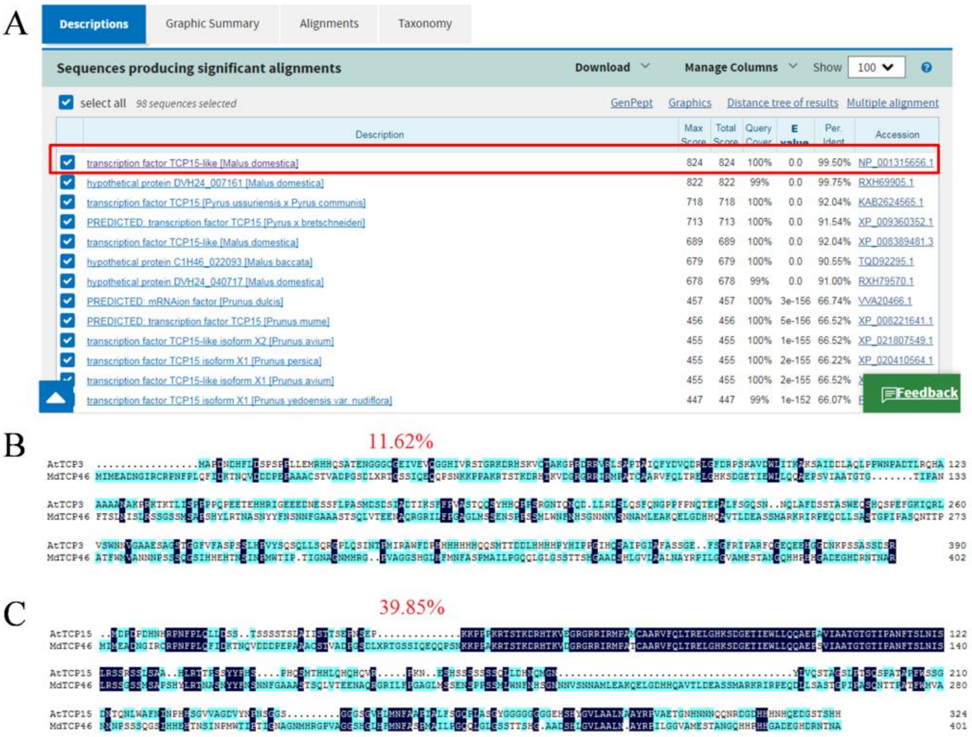

Supplemental Figure 4. MdTCP46 specifically interacts with MdMYB1 and MdBT2 in yeasts.

(a) MdTCP46 specifically interacted with MdMYB1 in yeasts. (b) MdTCP46 specifically interacted with MdBT2 in yeasts.

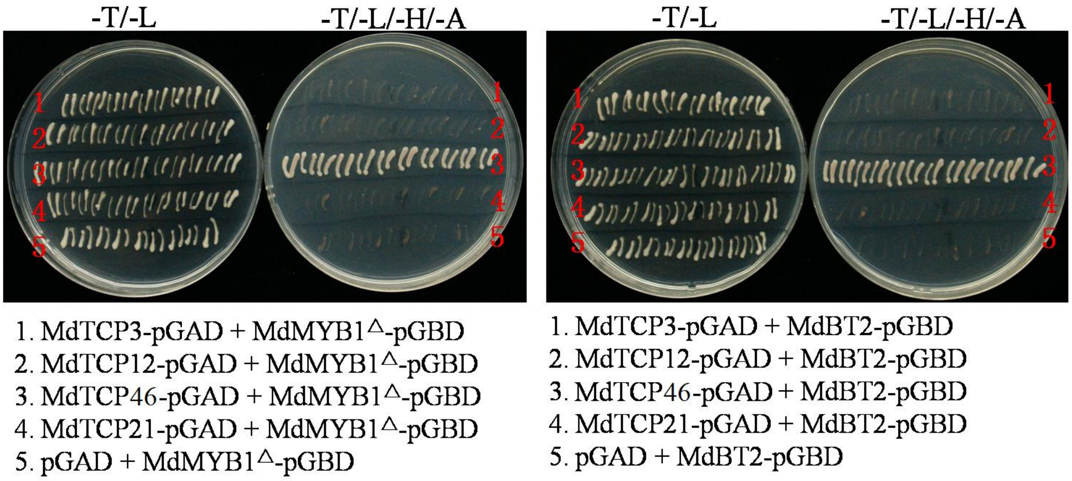

Supplement: eraa056_suppl_Supplementary_Figues_S1_S4 [file eraa056_suppl_supplementary_figues_s1_s4.pdf]
