# Supplementary material for: Dynamic regulation of anthocyanin biosynthesis at different light intensities by the BT2-TCP46-MYB1 module in apple
Source: J Exp Bot. 2020 Jan 30;71(10):3094–109. doi: 10.1093/jxb/eraa056 (PMC7475178; doi:10.1093/jxb/eraa056)
Supplement: eraa056_suppl_Supplementary_Tables_S1_S3 [file eraa056_suppl_supplementary_tables_s1_s3.pdf]

Supplementary Tables

Supplemental Table 1. The promoter sequence of *MdDFR*.

ATGTAATGAATAATATAAGAGAAAGGTAAAAATGTGATTTTTAGTTAAAA
GTGAACAGTAACGAGAGTGTTTCGTTAAAACTTTCTAAAAAATATACTCG
TGTTGATTCTCGAGTTCACATTTGGTCATTAAATTTCTTTGAATTATCAAAC
GAAACACCCAACCGTTTAATTCAGCAATATGCGCTCTACGATTTTTGGAAT
TTGAGAAGCCTATCTTGTGTGTGAGCACTGAAGAGCAAGGACAAAGACGT
AGGCATACCTCTCTATATACAAGTTTTGGGCCCATGGACATGGTTCTGTTC
TGTTTACCTATATGGATCGGCCAGCCCATAATACTTCGATTGATTTTGTAT
GAGTTTTGTTTCATATTTTCATTGAGAACATAGTCCAGCCCAATAACCAAG
TCCATCGCCATGGGCCCTTAGGCAACAAAATTTYMACTAAAACTCCCTAA
ATAAACCAGTTGTACTGGTGGATAGGCCTTAATCTTTAGAGTTCAATCAGA
ATGCCACGAACCACTCAGTATTTCAGTTTGATGATATTCTTCTTTACTTGA
AGTAAGAAGTTTTAGGTTTGAATCTCGTGAATGAAGAATACGACACCAAA
TTAGGTTGTCCATTATGTGGTTTAGCCGAACTCTCTCTTTCCTTAGTGTAAA
AATATCGATGTACTAAAAAAAAAAACTTAGTGTGCCTCTAGTTCAATTTA
ATATAGATATTCTTATAGTTTGTAAAAAAAAAAAAAAAAAAAATTGAACA
AACGATATTATTTACAATAAGGGAAAAGGGGGKGGGCTTAATCTTACAAT
GTGTTAGCAATAATGTGATTCGAATTCGCTTTTGGTGAGAATTGAACATAA
GACCTCTCACTTATAAGTGAAGGGGAATATCACTGAACCGTAGTACTAAG
TGGCTGTAAATATGTATTTTAAATTAGAGAAATGCTAAAGTACTCTTTCAA
AATTGGGACTCTTCATTGACTCTCCGCCACATCATGTCTCTGACACAATAT
TTTATAATGTTGTCACATAAATTGACGTTAAACTGTGAAATGATAGGGAGT
TCATAAATAAAGTTCCACATTTAAGAGAGTCTCATTAATATTTATGGATAA
ATTATGATTATATATTTATTGCTATTATCTTCGCCGCGCACAATTATTATTA
TCTATAACCATTACTATTTATCTGCTCGGCAATCGGCATGCCCAAACTGGT
TGTTGAAGGAAAATGAAGGGCTTTGACTTTGATTGAAGTGGTAGGGAACC
ATCGACTGTATCTGTGTTGAATTGGTTAGGTACCACCCAATAGGATAATTG
CTGACAAAAACACTCAAGAATTCTAATGCCACACGCACAATAGTTGAGGT
TAGTTGCTTGGCGTGACTTTATAAATGACACAAATACTATTAGCATAATGC
TGAAAAGACCAGTTAAAATATATCAGACTTTGATTTAAGATAAAGTAGAA
AATCTCCTATTTTTAACTTAGTTAAGTTAGTGTTGAACTTGAACGATTAGG
TTTTCGAGCCCATTGTCGTTAGACAAAAGTATCACTCTTGATTGCATGACT
TGAAAAATGCCGGGGACTTCGGGCGTTACTTTTGAAGTGCCGGTGACTTT
GGGCGTTATTTACAAAATGCCGGGGGACTTTGGGTGTCATTTTATGTTCGA
CGACTTTTGTTTTTTGGGGTGAATTTGTTTTCACGAACTAGAAAGTTGAGC
AGGTAACAAATGAAGTCAAGAACTAGGAAATCAACTACCACGCACGTGC
GTCAACCGTTAGGAAAGCACGTGATGCCCTGGAAGCTGAGATAAACAGA
GAAAGCAGGCGTGAGCAGGTTAGGCCACCAGTGTTAGCTGGGGCCGCAG
CACTCCACCAGTCCCCCACTATAAATATTTTATAAGCTTCCCCAGTCCTTC
ACGGAGCAACTATTCCAAGTACCTATTTTCCATCGATTTTTCAAGAGATAT
ACTTCGGTAAGCACATACACACAAGAT

Note: boxed nucleotides indicate the MdMYB1 binding site.

Supplemental Table 2. The promoter sequence of *MdUF3GT*.

ATGTCAGAATATTTCTGAGTCCCCTGTACCTTCATGTTCTTCTGGGACCTG
TTGCCCATTCCCTAGATTGACTGTTGGCCTGGGTCGTGGGAGAGGACCGA
GTCTTTCAGAAACTCTTGGGTCATTGATCTTCGAGCTTATGTGGATGGGAT
TCTCTCGACGTTGCTTTAGGAAGTCTCGACAATCGCGATAGACGACTTTTG
ATCCTTCCACTCCTTCTGTGAGGAGGTGCTTTCCTCTACTCCTTCTGCTTCG
GGTTGAAGCAGCTGGGTTGAGAGAAGTCTCATGTTGATTATCACATTGAT
GTGTAGCTCGATCCCTATCAGGGATGTCCGTGTTGGATATCGGTGACCCTC
CGTGTTGGGGGGCATTCAGATGATTGTTGACCTCAGCAGGGGCAACGAGC
TTGTGTGCTTAAGCATGCCTAGTCTCGTGAAGCATCTCGAAAAGTTTTTCA
TACTGCTCCTGGAGGACCTCGTTCCTCATCACTATCTTGTTGTTCTAAGCTT
CTAACTCATCGACTTTAGCTTGAAGAGTAACTCTATTTCCTTCCTGCTTTCG
TTGCTTCATACTAGGTCCAATGGAGGTGTCATTCTGTGTGTTATGGCTTCTT
TCGCTCCCCATGTTGGAAAGAGATGCTTGGGCAAAAGAAAGTGTACGAGC
GGTGGAAACCAGCTTGACAAAGCTGAAGAGAGTGAGAGTAAGTGTCTTTC
CCACAGACGGCGCCAAATGTTGATGCACAAAATCAGCGAGGACTTTGGTA
CAACAGAAAGTGTCAGGTTTGTGACCTTCGCTTGGTTGCTTCGATCACTAG
TGAAGATAAGTACGTAAATGAATAGGGACAGGGAAGCAAACACAAGATG
TACATGGTTCACCCAGATTGGCTACGTCCACGGAGTAGAGGAGTTCTCAT
TAATTGTGAAGGGTTTACACAAATACATAGGTTCAAGCTCTCCTTTTATGA
GTTCTAGTGAATAGTGTAGTACAAAATGACATTAGAGATTATTGTGGGAG
AATGATCCCTATTTATAGAAGAGAGTTTCTAGTTTTGTTCTGACATTGACA
TGTGTCGTGTTGTGATTGGCTTCTGATGTTGACACGTGTCGCGCTGTGGTT
GGCTTCTGATGTCGACACGTGTCGCATTGTGATTGACCTCCTGGTTGAAGG
GAAGCTCTTCTGGGTCATTGACGGTATAACGTTGATCGGTGCTCAGTAGTT
TCGGGATTGGTCAAGTATGGTACAAACAACTGCAATAAAATATTGCCACC
CCTAGGCCCTAGTCAAATAATAATGAGAACTTTAACGAAAAGCTCTCAGT
ACTGTTTACTTTAACAAAAAACCATATTTTTACACTAAAAAGTCAATCATG
ATACTATTCATTTTACCATTTATTTTATCTTTTATCTTTAAAACTCAAAGTT
TTCAAACAATTTTCATTAGTTTTCCTAATAATAATTCGGTGGGCTCCCCGG
GGTTGTAACGTGCATTGTTATTTGCTAATATCTAATGTAACATCCCACATC
GATAAACGGAAAGGGGGTGATGTGCCTTATATGTACATGCCCACCTTCAT
ATAGCACAACGCTTTTTGGGAACTCACTGATTTCGGGTTCCATCGTAACTC
CGAAGTTAAGCGAGTTCGGGTTGAGCATTCCCACGATGGGTGATTAATTG
GGAAGTTCTCATCTGAGTTCTCAGAAAAAAAAACCGTGAGCCTGTGGCCG
TGACCCAAAGCGGACAATATCGTGCTAAAATTGGGATGCGACATCTAACG
ATAACCATAGCAACCGGTCGGTCACCTCGTGCTGACTACCTTCCCGTCAGT
TATTGCAGGTGGTGACTGGTGAGTAGCGCATGCATTTCTTGTTAACGACA
AAGCAACGACTATATGAAGTCCTGTCCACTCCAGTTCAGTCCAATCCAAC
CTTAGCTAACTCCATTATTCCATCAGTACTGCTACTTCTATTCAACTCCTTT
TCTAATTAGCCTTGTAAGCTGTA

Note: boxed nucleotides indicate the MdMYB1 binding site.

Supplemental Table 3. Primers used for gene expression analysis and vector construction.

Primer name	sequence (5' to 3')
MdTCP46-F	ATGATCATGGAAGCAGATAA
MdTCP46-R	CCTAGCATTGGTATTACGAT
MdTCP46-Anti-F	AGGAGCTCCGGGTCCAGCAT
MdTCP46-Anti-R	ATTAATGGAATTAGTGTGCT
MdTCP46(qRT)-F	AGGAGCTCCGGGTCCAGCAT
MdTCP46(qRT)-R	ATTAATGGAATTAGTGTGCT
MdMYB1-F	ATGGAGGGATATAACGAAA
MdMYB1-R	TTCTTCTTTTGAATGATTCC
MdMYB1(qRT)-F	AAGACCTCAGCCCCAAAAATTCAA
MdMYB1(qRT)-R	CTTTTGAATGATTCCAAAGGTCCG
MdDFR(qRT)-F	GTTGAGGGAGATAGGGTTTGAG
MdDFR(qRT)-R	GGTAAATGTAAAACAATAGAGAGG
MdUF3GT(qRT)-F	GGAAGTGGTTTTGTCGCCTG
MdUF3GT(qRT)-R	CATTATTATTGAGCAACGAACAGC
MdCHI(qRT)-F	GCTACAAATGCGGTGATAG
MdCHI(qRT)-R	CGCCTCCACTACAACCTCC
MdCHS(qRT)-F	GGCAAGTGCTGTCGGATT
MdCHS(qRT)-R	CCCAAAGAAATAACCACAAG
MdDFR-Probe-F	CGTGCGTCAACCGTTAGGAAAGCACG
MdDFR-Probe-R	CGTGCTTTCCTAACGGTTGACGCACG
MdDFR-Probe-Mut-F	CGTGCGTCAAGCCAGGGGAAAGCACG
MdDFR-Probe-Mut-R	CGTGCTTTCCCCTGGCTTGACGCACG
MdDFR-LUC-F	CGTCAACCGTTAGGAAAG
MdDFR-LUC-R	CTTGTGTGTATGTGCTTACC
MdUF3GT-Probe-F	ACCTTCCCGTCAGTTATTGCAGGTGG
MdUF3GT-Probe-R	CCACCTGCAATAACTGACGGGAAGGT
MdUF3GT-Probe-Mut-F	ACCTTCCCGTGCCAGGTTGCAGGTGG
MdUF3GT-Probe-Mut-R	CCACCTGCAACCTGGCACGGGAAGGT
MdUF3GT-LUC-F	TCCCGTGCCAGGTTGCAG
MdUF3GT-LUC-R	CAGCTTACAAGGCTAATTAG
MdBT2-F	ATGGAAGCTAATCCGACCGCAA
MdBT2-R	TCACAATCTGAAGCTTCTAAT
MdBT2-Anti-F	ATTCTTGCAAGCTCACGACCCTTG
MdBT2-Anti-R	ATCTGGAGGCCTTGACACGTG
MdBT2(qRT)-F	GCAACTGGCGAAGATGTGCGAT
MdBT2(qRT)-R	CTTCTTGTAACCTGGCTCCATG
MdTCP3-F	ATGCATCAAACAAATGAACG
MdTCP3-R	ATGGCGAGAATTGGAGGAAG
MdTCP12-F	ATGTTTCCTTCTAGCAGCAC
MdTCP12-R	ATTTCCAGTAAACAGGGGCT
MdTCP21-F	ATGTCAAATTCCGAGGACCC
MdTCP21-R	GCGCCGGTCGTCTTCTCTCT
